# Rap1 deficiency-provoked paracrine dysfunction impairs immunosuppressive potency of mesenchymal stem cells in allograft rejection of heart transplantation

**DOI:** 10.1038/s41419-018-0414-3

**Published:** 2018-03-07

**Authors:** Yue Ding, Xiaoting Liang, Yuelin Zhang, Li Yi, Ho Cheung Shum, Qiulan Chen, Barbara P. Chan, Huimin Fan, Zhongmin Liu, Vinay Tergaonkar, Zhongquan Qi, Hung-fat Tse, Qizhou Lian

**Affiliations:** 1 Department of Organ Transplantation, Changzheng Hospital, Second Military Medical University, Shanghai, China; 2 Department of Medicine, The University of Hong Kong, Hong Kong SAR, China; 3 Organ Transplantation Institute of Xiamen University, Xiamen, Fujian Province China; 4 Translational Medical Center for Stem Cell Therapy, Shanghai East Hospital, Tongji University School of Medicine, Shanghai, China; 5 Clinical Translational Medical Research Center, Shanghai East Hospital, Tongji University School of Medicine, Shanghai, China; 6 Department of Emergency, Guangdong General Hospital, Guangdong Academy of Medical Sciences, Guangzhou, China; 7 Peking University Shenzhen Hospital, Shenzhen, China; 8 Department of Mechanical Engineering, The University of Hong Kong, Hong Kong SAR, China; 9 Department of Cardiovascular and Thoracic Surgery, Shanghai East Hospital, Tongji University School of Medicine, Shanghai, China; 10 Institute of Molecular and Cellular Biology, Biopolis, Singapore; 11 School of Biomedical Sciences, The University of Hong Kong, Hong Kong SAR, China

## Abstract

Immunomodulatory activity of mesenchymal stem cells (MSCs) is largely mediated by paracrine factors. Our previous studies showed that activation of nuclear factor-kappa B (NF-κB) regulates cytokine/growth factor secretion by MSCs. This study aimed to elucidate the role of Rap1 (repressor/activator protein), a novel modulator involved in the NF-κB pathway, in regulating the immunomodulatory potency of MSCs in acute allograft rejection of heart transplantation. The immunosuppressive potency of wild-type MSCs (WT-MSCs) or Rap1-deficient MSCs (Rap1^−/−^-MSCs) was examined in mice with acute allograft rejection following heart transplantation. With a combination of immunosuppressant rapamycin at a dose of 1 mg/kg/d, WT-MSCs notably prolonged the survival of the transplanted heart compared with Rap1^−/−^-MSCs. Rap1^−/−^-MSCs displayed a marked insensitivity to inhibit the mixed lymphocyte reaction (MLR) due to impaired cytokine production and a significantly reduced activity of NF-κB signaling *in vitro*. Finally, transplantation of encapsulated WT-MSCs greatly prolonged the survival of the heart allograft compared with encapsulated Rap1^−/−^-MSCs. Our results indicate that Rap1 is essential to maintain the immunomodulatory function of MSCs. Deletion of Rap1 results in impaired immunomodulatory function of MSCs.

## Introduction

Mesenchymal stem cells (MSCs) have been used to regulate the innate and adaptive immune system as a potential approach to prevent or treat allograft rejection following kidney transplantation^1^, hematopoietic stem cell transplantation^2^, and in animal models of allogeneic heart and islet transplantation^3,4^. Nonetheless, knowledge of the immunosuppressive capacity of MSCs remains limited. Bone marrow MSCs fail to prevent graft-versus-host disease (GVHD) in mice although they suppress lymphocyte proliferation *in vitro*^5^. In clinical trials, application of MSCs for therapy-resistant GVHD showed moderate or even worsened efficacy in a subset of patients^6^. Recent studies have demonstrated that the immunosuppressive ability of MSCs is not constitutive but inducible. Li and colleagues proposed that the degree of nitric oxide production elicited by proinflammatory cytokines in the surrounding environment influenced MSCs to be either potently immunosuppressive or highly immune enhancing^7^. Thus, to guarantee a most effective activation of their immunosuppressive characteristics, more detailed information about the mechanisms and signaling pathways may help to harness MSC as an immunoregulator.

Although the potential mechanisms underlying MSC-mediated immunomodulation are not fully understood, accumulating evidence demonstrates that production of soluble factors is predominately responsible^8^. The nuclear factor-kappa B (NF-κB) family of transcription factors plays a critical role in coordinating the expression of a wide variety of inflammatory-related genes, including cytokines, chemokines, and adhesion molecules, to regulate immune responses^9^. It has been documented that NF-κB activation mediates cytokine/growth factor secretion by MSCs^10^. It is thus reasonable to postulate that intervention with NF-κB to regulate its paracrine capacity can affect the immunoregulatory effects of MSCs.

Recently, we performed a genome-wide gain-of-function screen and unexpectedly identified a telomeric protein Rap1 (repressor/activator protein) as an IκB kinase (IKK) adapter that modulates NF-κB in a dose-dependent manner^11^. Rap1-mutant mice showed a defective NF-κB signaling and reduced the generation of responding cytokines when stimulated by lipopolysaccharides (LPS)^11^. Whether Rap1 is involved in regulating the immunomodulatory potential of MSCs has not been investigated. In this study, we aimed to determine whether inhibition of Rap1 could affect the immunomodulatory potential of MSCs by regulating the responding cytokine profiling.

## Results

### Characterization of Rap1^−/−^-MSCs

**Fig. 1:**
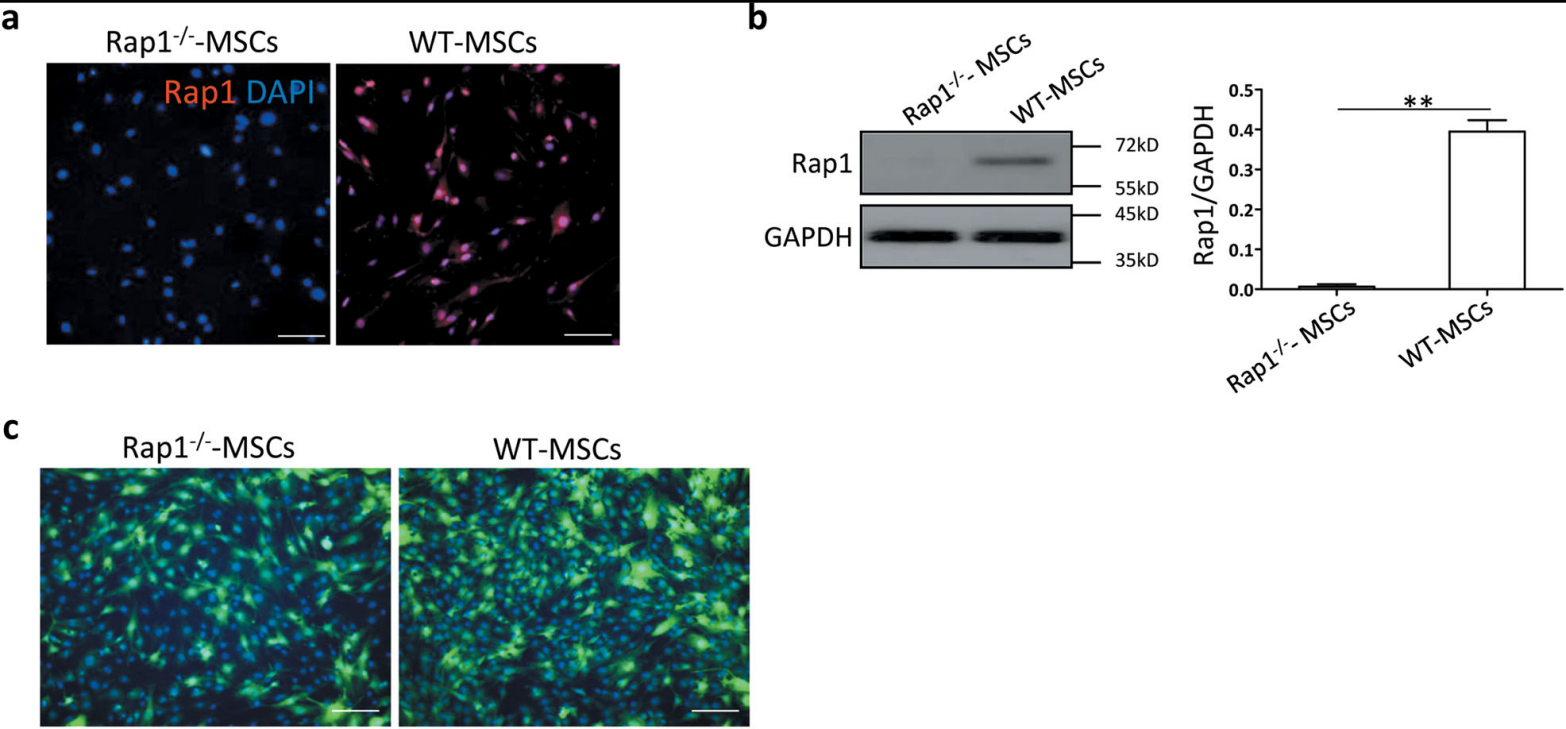
Lentiviral GFP labeling of MSCs. Rap1 expression was negative in Rap1^−/−^-MSCs but positive in WT-MSCs, examined by immunostaining (**a**) and western blotting (**b**), respectively. **c** Rap1^−/−^-MSCs and WT-MSCs were successfully infected with lentiviral GFP, detected by immunostaining. N: non-significance. Scale bar = 200 μM.

First, the genotyping of Rap1 knockout (Rap1^−/−^) and wild-type (WT) mice was performed as previously described^11,12^. MSCs isolated from tibias were cultured until passage 4 to exclude non-adherent hematopoietic cells. Similar to wild-type MSCs (WT-MSCs), MSC surface markers were positive for CD105, CD73, CD90, CD44, Sca1, and not contaminated for CD34 or CD45 positive hematopoietic cells in Rap1 knockout MSCs (Rap1^−/−^-MSCs) (Supplementary Fig. 1A). The multiple differentiation potential for adipogenesis, chondrogenesis, and osteogenesis was not affected by Rap1 knockout (Supplementary Fig. 1B). Moreover, Rap1 deficiency in MSCs did not affect their proliferation *in vitro* (Supplementary Fig. 1C). Rap1 expression was negative in Rap1^−/−^-MSCs and positive in WT-MSCs, examined by immunostaining (Fig. 1a) and western blotting (Fig. 1b), respectively. Furthermore, MSCs were successfully labeled with green fluorescent protein (GFP) by lentiviral infection, confirmed by immunofluorescence (Fig. 1c).

### Rap1 activates NF-κB transcriptional activity in MSCs

**Fig. 2:**
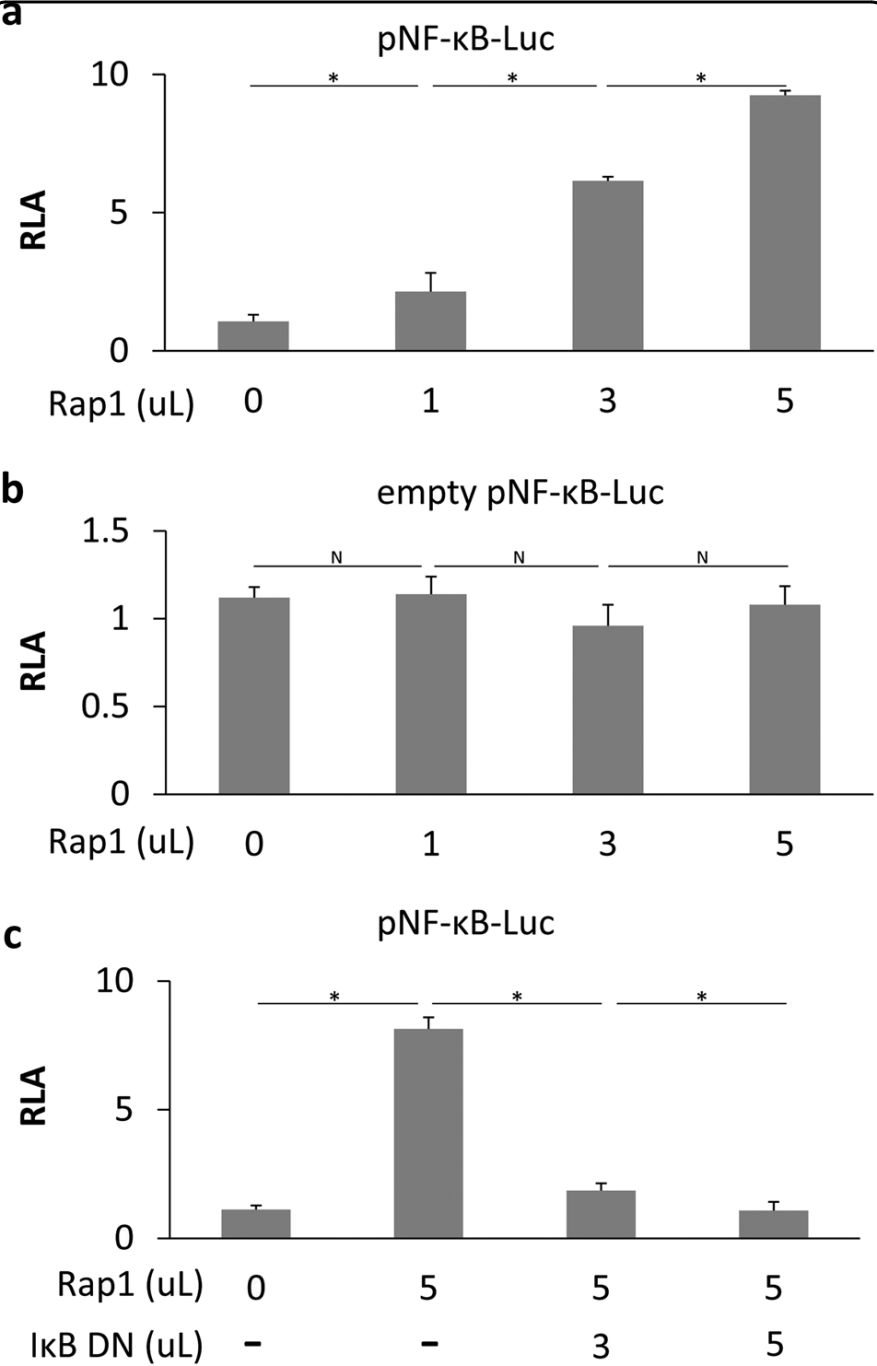
Rap1 activates NF-κB transcriptional activity. **a** Rap1^−/−^-MSCs were transfected with pNF-κB luciferase reporter pNFκB-Luc. **b** Transfection of an empty pNF-κB-Luc was set as controls. The amounts of expression plasmid for Rap1 (**a**, **b**) and IκB DN (**c**) were progressively increased as indicated from 1 to 5 µL (60 ng of DNA/µL). RLA was expressed as firefly luciferase activity after it was normalized to Renilla luciferase activity (*n* = 3 for each group). *p < 0.05; N: non-significance, IκB DN: IκB-dominant negative, RLA: relative luciferase activity.

The relationship between NF-κB and Rap1 was examined *in vitro*. A gene construct pNF-κB-Luc carrying luciferase as a reporter was used to examine the cellular activities of the corresponding signaling pathways. This construct contains a phosphorylated NF-κB (pNF-κB) enhancer element located upstream of the secreted luciferase gene. Binding of transcription factors to the pNF-κB enhancer element allows luciferase to be expressed and secreted into the surrounding medium. As shown in Fig. 2, Rap1^−/−^-MSCs were transfected with the indicated plasmids. Groups A and C received reporter plasmid pNF-κB-Luc, whereas group B received empty pNF-κB-Luc as controls. The amount of expression plasmid for Rap1 (Fig. 2a, b) and IκB-dominant negative (IκB DN) (Fig. 2c) was progressively increased as indicated from 1 to 5 µL (60 ng of DNA/µL). Compared with empty controls (Fig. 2b), pNF-κB activation was gradually enhanced along with an increasing amount of Rap1 plamids (Fig. 2a). In contrast, overexpression of IκB DN largely abolished Rap1-activated pNF-κB-Luc activity (Fig. 2c). These results showed that the activity of NF-κB transcriptional signaling was activated by Rap1.

### Impaired immunomodulatory functions of Rap1^−/−^-MSCs in allogeneic heart transplantation

**Fig. 3:**
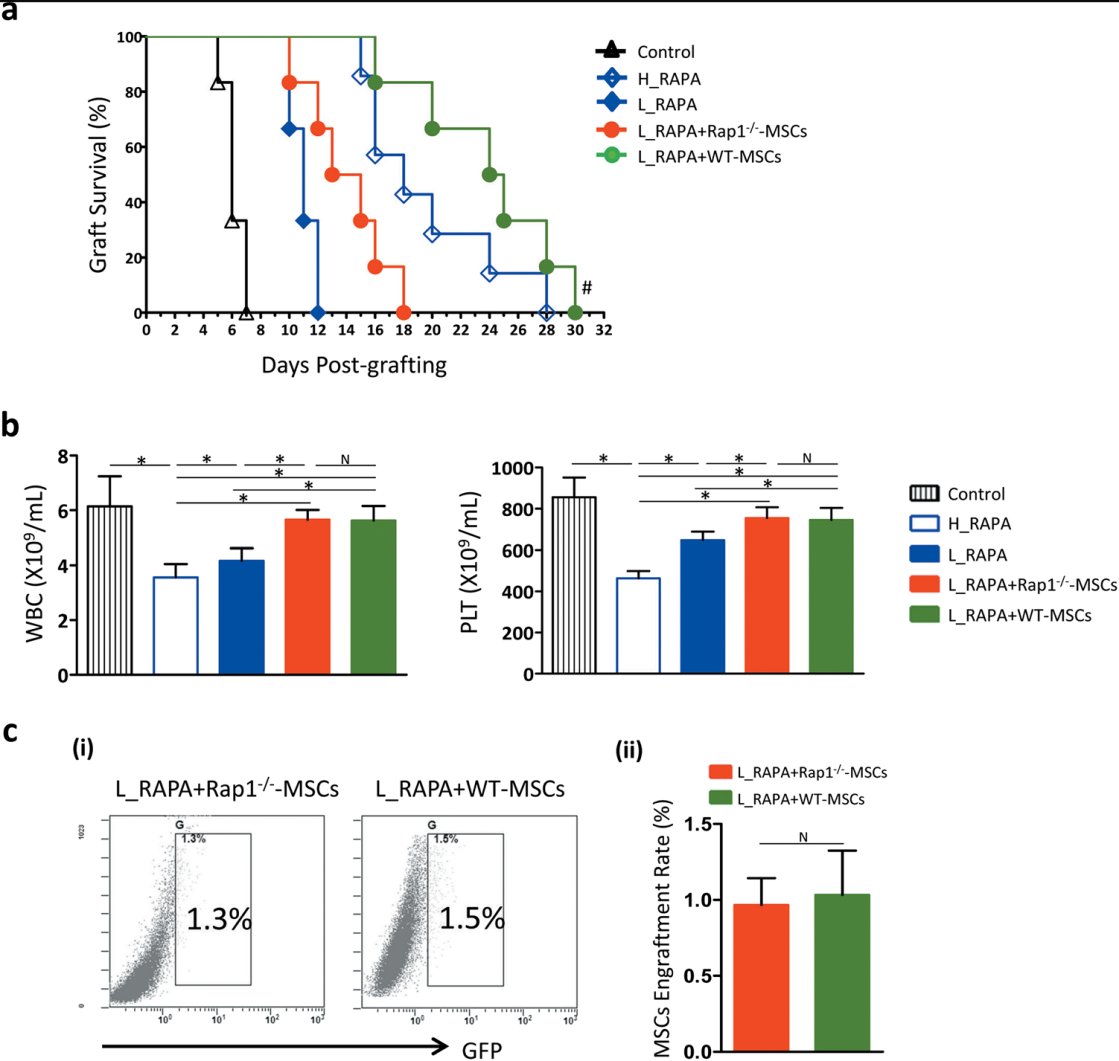
Rap1 deficiency in MSCs impairs their ability as immune adjuvants of rapamycin. **a** Graft survival was shown by Kaplan–Meier method and compared by log-rank test (*n* = 16 for each group). ^#^*p* < 0.05 *vs*. L_RAPA + Rap1^−/−^-MSC group. **b** WBC and PLT count were measured at 48 h after transplantation (*n* = 6 for each group). **c** Cell engraftment in the transplanted heart was determined by GFP-positive cell sorting (*n* = 3 for each group). *p < 0.05; N: non-significance, WBC: white blood cell, PLT: platelet.

To examine whether Rap1 deficiency in MSCs leads to an impaired immunomodulatory potency *in vivo*, the recipient C57/B6 mice were treated with Rap1^−/−^-MSCs/WT-MSCs combined with rapamycin (RAPA) at a dose of 1 mg/kg/d as described previously^4,13^. The median survival time (MST) of transplanted hearts treated with a high dose of rapamycin (H_RAPA) was 18 days (H_RAPA group, Fig. 3a), compared with 11 days in the low-dose group (L_RAPA group, Fig. 3a). Infusion of MSCs and a low dosage of RAPA was synergistic in promoting cardiac allograft tolerance, especially in the L_RAPA + WT-MSC group where MST was prolonged to 24.5 days (Fig. 3a), compared with 14 days in the L_RAPA + Rap1^−/−^-MSC group (Fig. 3a, *p* < 0.05). We also examined platelet (PLT) and white blood cell (WBC) counts at 48 h after surgery because a decrease in these two variables is a primary toxic side effect of RAPA^14^. PLT and WBC counts showed a dose-dependent toxicity during RAPA treatment, and Rap1^−/−^-MSCs and WT-MSCs were equally capable of reversing the downtrend (Fig. 3b). To quantify the survival rate of transplanted cells, cardiac allografts were digested at 48 h after transplantation. The result showed that very few GFP^+^ cells were detected (Fig. 3c-i&ii).

### Combination of rapamycin and WT-MSCs, but not Rap1^−/−^-MSCs, more effectively prevents inflammation and induces differential regulation of tolerance-associated CD4^+^Foxp3^+^ regulatory T cells in the cardiac allograft

**Fig. 4:**
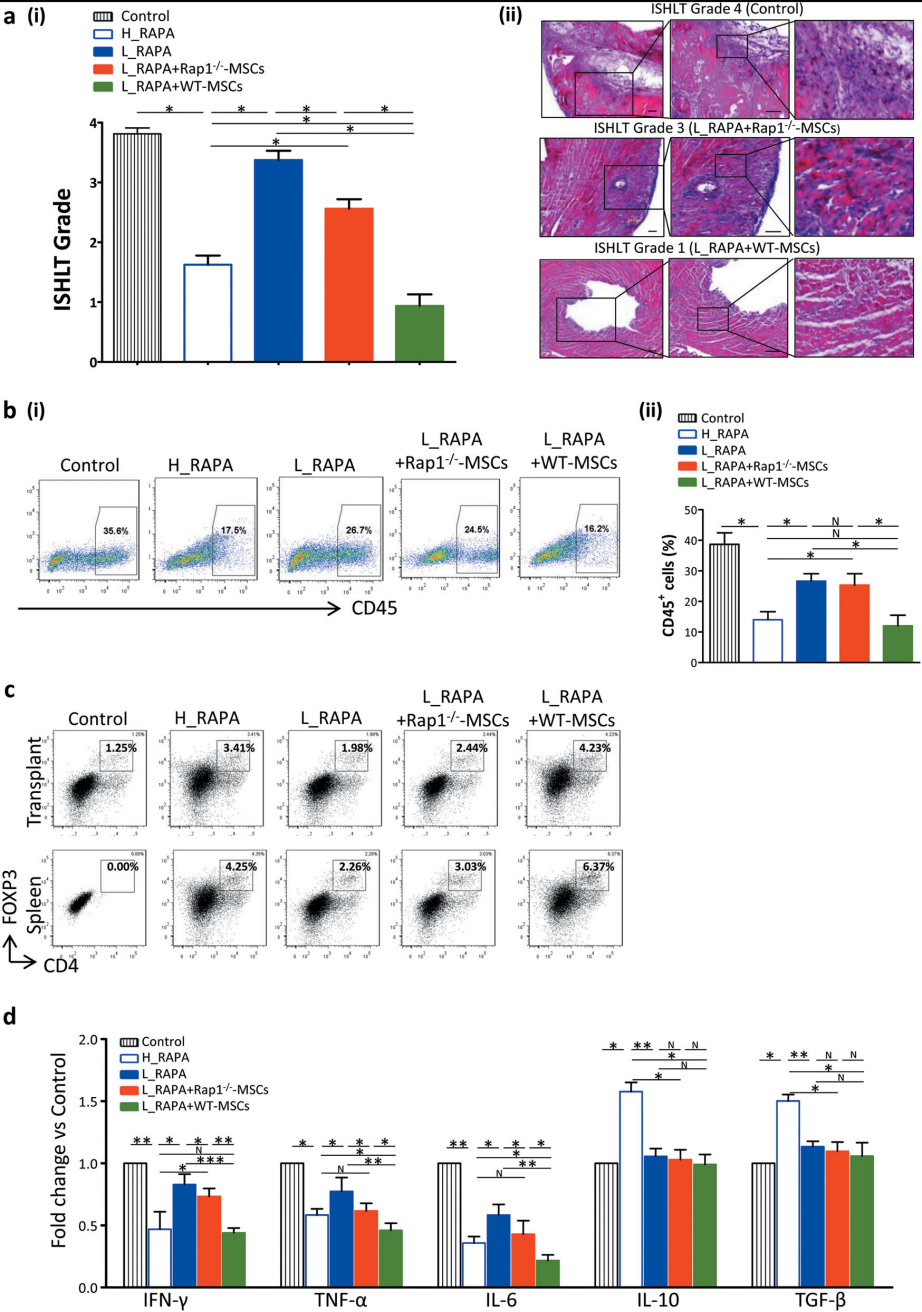
Rap1 deficiency decreases the immunosuppressive ability of MSCs *in vivo*. **a** Representative photographs showing the ISHLT score for each group (i) and typical H&E staining of grafts (ii) (*n* = 6 for each group). Scale bar = 100 μM. **b** Quantification of infiltrating CD45^+^ mononucleocyte population in cells isolated from transplanted hearts at 48 h after surgery by FACS analysis (*n* = 3 for each group). **c** Proportions of splenic and allograft transplant CD4^+^Foxp3^+^ Treg cells were analyzed by flow cytometry at day 2 post transplantation (*n* = 3 for each group). **d** The concentration of IFN-γ, TNF-α, IL-6, IL-10, and TFG-β in sera from rejected hearts was measured by Flowcytomix (*n* = 10 for each group). *p < 0.05; **p < 0.01; ***p < 0.001; N: non-significance.

To evaluate the severity of inflammatory infiltration following transplantation, the corresponding ISHLT (International Society of Heart and Lung Transplantation) score of the hearts harvested from the different treatment groups was assessed as previously described^15^. The myocardium of the grafts from the control group (ISHLT Grade 4) showed extensive necrosis and infiltration of inflammatory cells (Fig. 4a-i&ii). However, the grafts from the H_RAPA, L_RAPA, and L_RAPA + Rap1^−/−^-MSC groups (ISHLT Grade 3) showed moderate lymphocyte infiltration and tissue damage (Fig. 4a-i&ii). L_RAPA + WT-MSC group (ISHLT Grade 1) presented the lowest level of lymphocytic infiltration and fewest changes in myocardial structural integrity among all groups (Fig. 4a-i&ii). The inflammatory monocyte population of the allograft was analyzed by flow cytometry at 48 h after transplantation. RAPA reduced the percentage of CD45^+^ cells in a dose-dependent manner (Fig. 4b-i&ii). Notably, WT-MSCs were more capable in suppressing CD45^+^ inflammatory cell infiltration than Rap1^−/−^-MSCs (Fig. 4b-i&ii). Lymphocytes separated from spleens and allograft transplants of recipient mice were analyzed by FACS (fluorescence-activated cell sorter) at 48 h after transplantation. Compared with the L_RAPA + Rap1^−/−^-MSC group, the proportion of CD4^+^FOXP3^+^ regulatory T cells (Tregs) was significantly increased in the L_RAPA + WT-MSC group (Fig. 4c). To evaluate the inflammation status systematically, we collected the serum from cardiac puncture and performed cytokine profiling at 48 h after transplantation. L_RAPA + Rap1^−/−^-MSC and L_RAPA + WT-MSC treatment groups had a comparable level of anti-inflammatory cytokine IL-10 (interleukin 10) and TGF-β (transforming growth factor β), but proinflammatory cytokines IFN-γ (interferon-γ), TNF-α (tumor necrosis factor α), and IL-6 (interleukin 6) were significantly lower in the L_RAPA + WT-MSC group than those in the L_RAPA + Rap1^−/−^-MSC group (Fig. 4d). Collectively, the data suggested that WT-MSCs are superior to Rap1^−/−^-MSCs in ameliorating lymphocyte infiltration and inducing immune tolerance.

### Reduced potency of Rap1^−/−^-MSCs to inhibit MLR *in vitro* is mainly through impaired cytokine secretion, and not cell–cell contact

**Fig. 5:**
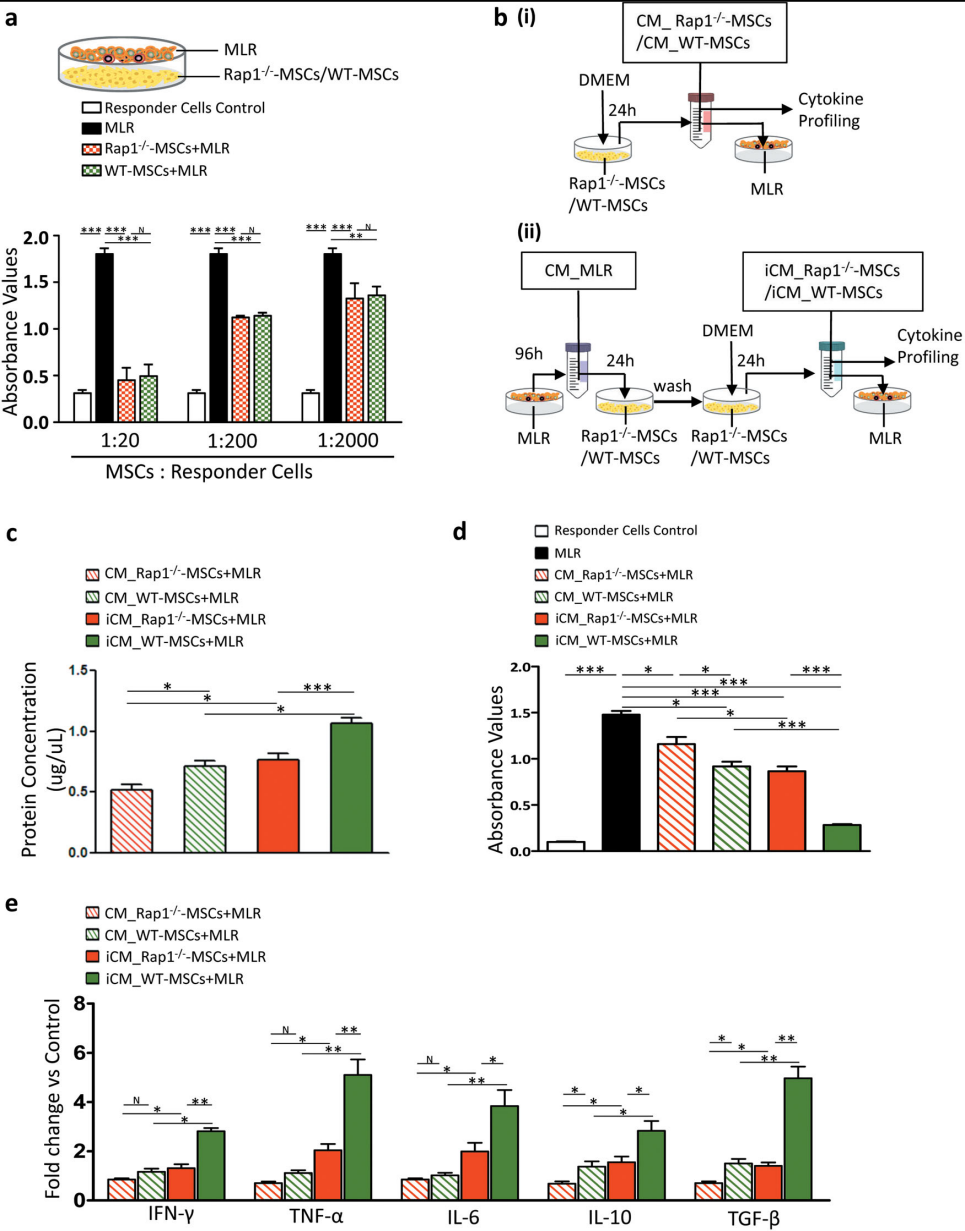
Rap1 deficiency impairs MSC paracrine action to suppress MLR. **a** WT-MSCs and Rap1^−/−^-MSCs showed a significant and comparable capacity to inhibit MLR in a coculture setting (*n* = 3 for each group). **b** Schematic diagram showing (i) the rest status of MSC-conditioned medium collection process and (ii) active status of MSC-conditioned medium collection process. **c** Differential concentration of secreted protein in the conditioned medium was determined among different stimulatory conditions (*n* = 3 for each group). **d** Conditioned medium from Rap1^−/−^-MSCs showed inferior capacity to suppress MLR compared with WT-MSCs (*n* = 3 for each group). **e** Cytokine profiling showed that Rap1 deficiency resulted in impaired cytokine secretion in MSCs, including proinflammatory cytokines (IFN-γ, TNF-α, and IL-6), and anti-inflammatory cytokines (IL-10 and TGF-β). *p < 0.05; **p < 0.01; ***p < 0.001; N: non-significance.

Immunomodulation of MSCs is believed to occur through MSC-immune cell contacts and/or MSC-secreted cytokines^16^. In a coculture setting, in which cell–cell communication and paracrine effects are involved, WT-MSCs and Rap1^−/−^-MSCs displayed a comparable ability to suppress the mixed lymphocyte reaction (MLR), and a gradual enhanced inhibition was observed in line with an increasing proportion of MSCs (Fig. 5a). We next investigated the role of paracrine effects in regulating MLR. At first, we compared the paracrine effects between WT-MSCs and Rap1^−/−^-MSCs, at rest status, on suppression of MLR. As shown in Fig. 5b-i, c, poorer concentrations of secreted proteins were observed in the conditioned medium of Rap1^−/−^-MSCs (CM_Rap1^−/−^-MSCs) compared with that in WT-MSCs (CM_WT-MSCs) (Fig. 5c, *p* < 0.05). Although both CM_Rap1^−/−^-MSCs and CM_WT-MSCs suppressed MLR, a much stronger inhibition was observed in CM_WT-MSCs (Fig. 5d, *p* < 0.05). Moreover, WT-MSCs exhibited a superior ability in cytokine secretion compared with Rap1^−/−^-MSCs, including proinflammatory factors (IFN-γ, TNF-α, and IL-6), and anti-inflammatory factors (IL-10 and TGF-β) (Fig. 5e).

Next, to understand paracrine effects of MSCs, at active status, on suppression of MLR, the conditioned medium collected from MLR (CM_MLR) was used as an inducer to activate Rap1^−/−^-MSCs and WT-MSCs, as shown in Fig. 5b-ii. After washing away of CM_MLR stimulation, the conditioned medium of MSCs was harvested for cytokine profiling and MLR experiment. It showed that, compared to rest status, CM_MLR treatment effectively increased protein yield in the conditioned medium of both Rap1^−/−^-MSCs and WT-MSCs (Fig. 5c, *p* < 0.05 *vs*. non-CM_MLR treatment) and enhanced their ability to suppress MLR (Fig. 5d, *p* < 0.05 *vs*. non-CM_MLR treatment).

CM_MLR treatment demonstrated its effectiveness in stimulating immunomodulatory cytokine secretion: CM_MLR induced Rap1^−/−^-MSCs (iCM_Rap1^−/−^-MSCs) and WT-MSCs (iCM_WT-MSCs) had a higher abundance of cytokines compared with their untreated counterparts (Fig. 5c), indicating that the immunomodulatory effects of MSCs are inducible. Importantly, Rap1^−/−^-MSCs exhibited a markedly reduced capacity to respond to CM_MLR stimulation compared with WT-MSCs, as evidenced by significantly decreased cytokine release, including proinflammatory cytokines (IFN-γ, TNF-α, and IL-6) and anti-inflammatory cytokines (IL-10 and TGF-β) (Fig. 5e).

### Absence of Rap1 downregulates NF-κB activity in MSCs

**Fig. 6:**
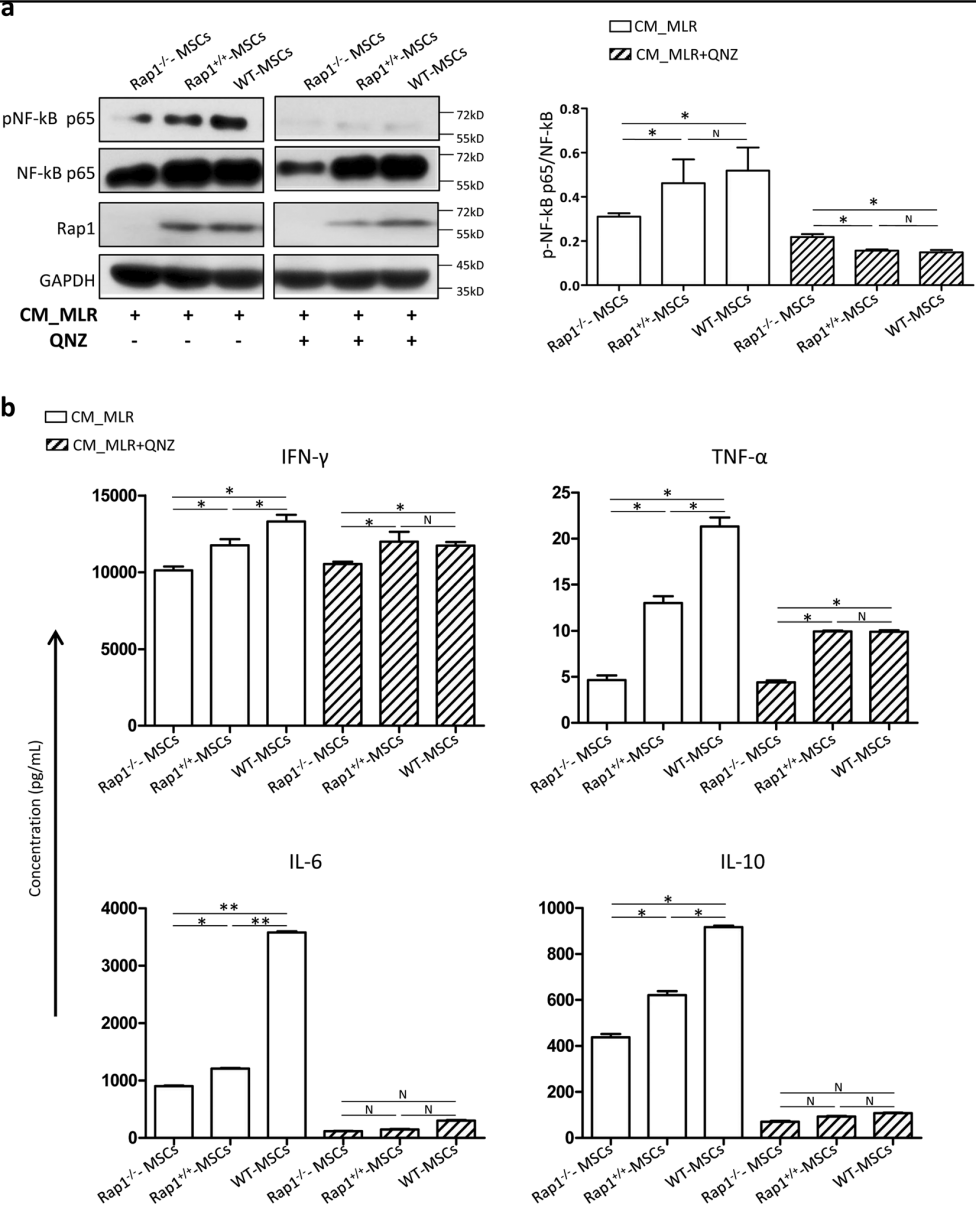
Absence of Rap1 downregulates NF-κB activity in MSCs. **a** Western blotting showed the activity of NF-κB pathway in active status with/without NF-κB inhibitor QNZ treatment. **b** Cytokine profiling showed that Rap1 deficiency downregulated NF-κB-related cytokine secretion in MSCs (*n* = 3 for each group). *p < 0.05; **p < 0.01; ***p < 0.001; N: non-significance.

We used lipofectin transfection to overexpress Rap1 into Rap1^−/−^-MSCs, and referred it as Rap1^+/+^-MSCs. Western blotting showed that Rap1 expression was successfully restored to a level comparable to WT-MSCs in Rap1^+/+^-MSCs (Fig. 6a). NF-κB p65 expression level was lower in Rap1^−/−^-MSCs compared with WT-MSCs, but its expression was restored in Rap1^+/+^-MSCs (Fig. 6a). A significantly higher ratio of pNF-κB p65/NF-κB p65 was detected in WT-MSCs compared with Rap1^−/−^-MSCs following CM_MLR treatment, and the trend was partially rescued by Rap1 overexpression (Fig. 6a). The activity of NF-κB was inhibited by NF-κB signaling inhibitor QNZ, demonstrated by a significantly lower ratio of pNF-κB p65/NF-κB p65, especially in WT-MSCs and Rap1^+/+^-MSCs (Fig. 6a). To determine whether downregulated NF-κB activity resulted in dysfunction of cytokine secretion in MSCs, we tested the secreted level of IFN-γ, TNF-α, IL-6, and IL-10, which are reported to be largely controlled by NF-κB signaling pathway^17–20^. Protein levels of IFN-γ, TNF-α, IL-6, and IL-10 were significantly lower in Rap1^−/−^-MSCs compared with those in WT-MSCs after CM_MLR treatment (Fig. 6b). Replenishing Rap1 expression in Rap1^−/−^-MSCs alleviated secretion of the above cytokines to a level comparable to WT-MSCs (Fig. 6b). NF-κB inhibitor QNZ significantly reduced the above cytokine secretion (Fig. 6b). Rap1^+/+^-MSCs demonstrated a better capacity in paracrine function compared to Rap1^−/−^-MSCs, but not as capable as WT-MSCs (Fig. 6b). Collectively, the absence of Rap1 impairs NF-κB activity and results in dysfunction of cytokine secretion in MSCs.

### Transplantation of encapsulated WT-MSCs more effectively prolongs the survival of the allogeneic heart than Rap1^−/−^-MSCs

**Fig. 7:**
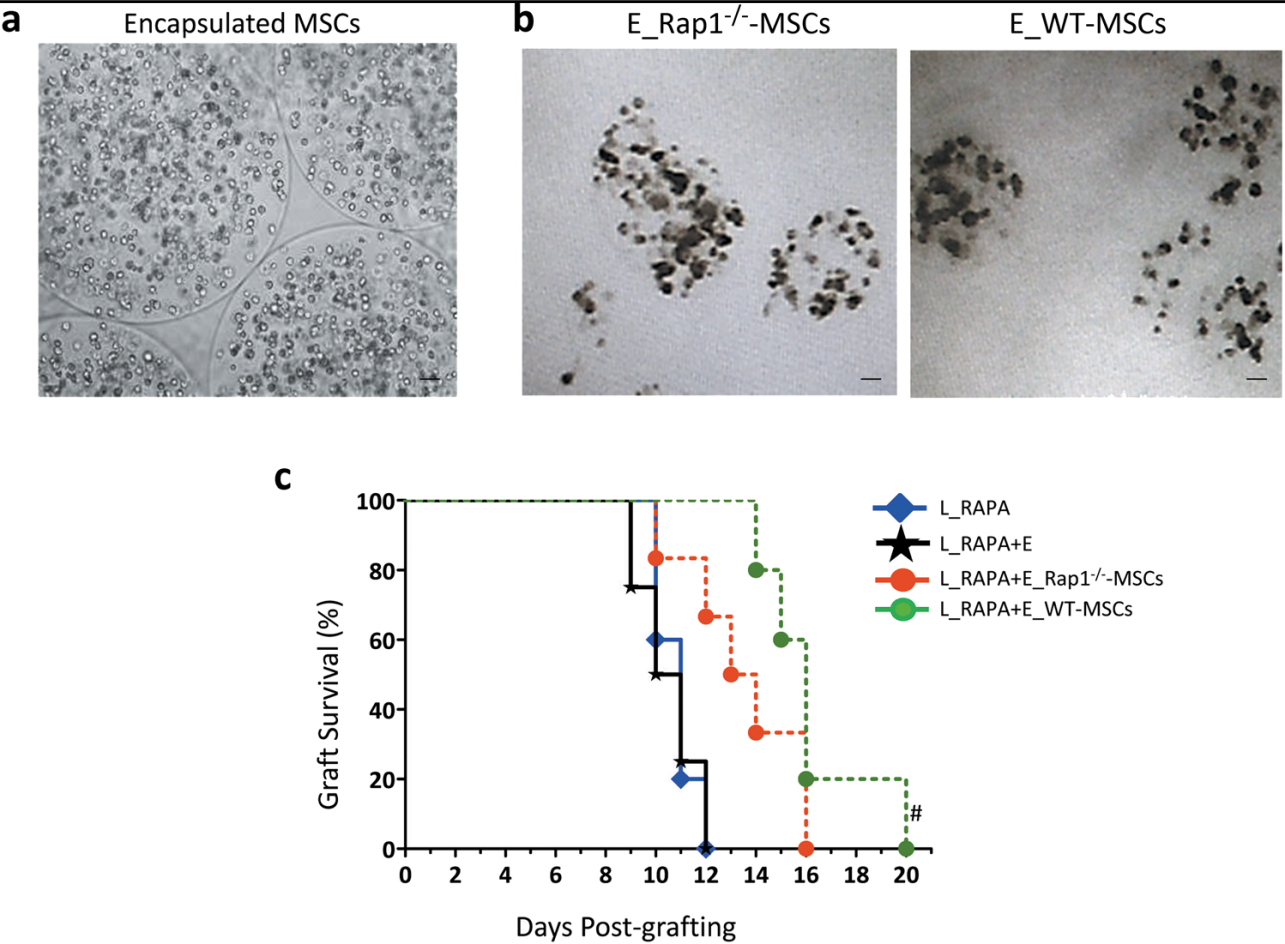
E_WT-MSCs more effectively extend allograft survival than E_Rap1^−/−^-MSCs as immune adjuvants of rapamycin. **a** Morphological presentation of newly made encapsulated MSCs. **b** Encapsulated Rap1^−/−^-MSCs/WT-MSCs after 10 days of culture. **c** Graft survival was shown by Kaplan–Meier method and compared by log-rank test (*n* = 10 for each group). ^#^*p* < 0.05 *vs*. L_RAPA + E_Rap1^−/−^-MSC group. Scale bar = 200 μM.

We have demonstrated that Rap1^−/−^-MSCs have distinct cytokine secretion compared to WT-MSCs (Fig. 5e), and cell engraftment was barely detected in the cardiac allograft (Fig. 3c), suggesting that the paracrine factors might act predominantly in regulating allogeneic rejection. To allow analysis of the secreted factors from MSCs in the regulation of allograft survival, we used sodium alginate to encapsulate Rap1^−/−^-MSCs and WT-MSCs (Fig. 7a), in which cells are able to survive and release cytokines dynamically without direct exotic–autologous contacts^21^. The encapsulated MSCs were able to survive *in vitro* for about 10 days (Fig. 7b). The encapsulated Rap1^−/−^-MSCs (E_Rap1^−/−^-MSCs) or encapsulated WT-MSCs (E_WT-MSCs) were intraperitoneally infused into mice that underwent heart transplantation. RAPA was applied as the dominant immunosuppressant and E_Rap1^−/−^-MSCs or E_WT-MSCs functioned as an immunological adjuvant. In agreement with the outcome of direct Rap1^−/−^-MSC/WT-MSC treatment (Fig. 3a), the combination of E_WT-MSCs and RAPA treatment achieved a longer allograft survival than E_Rap1^−/−^-MSCs (Fig. 7c), suggesting that the cytokines released from MSCs are involved in regulating allograft rejection. Nonetheless, although the tendencies were generally the same, the effects of encapsulated MSCs were weaker than direct cell injection, as shown by a relatively shorter survival time of the allografts (Fig. 3a *vs*. Fig. 7c).

## Discussion

**Fig. 8:**
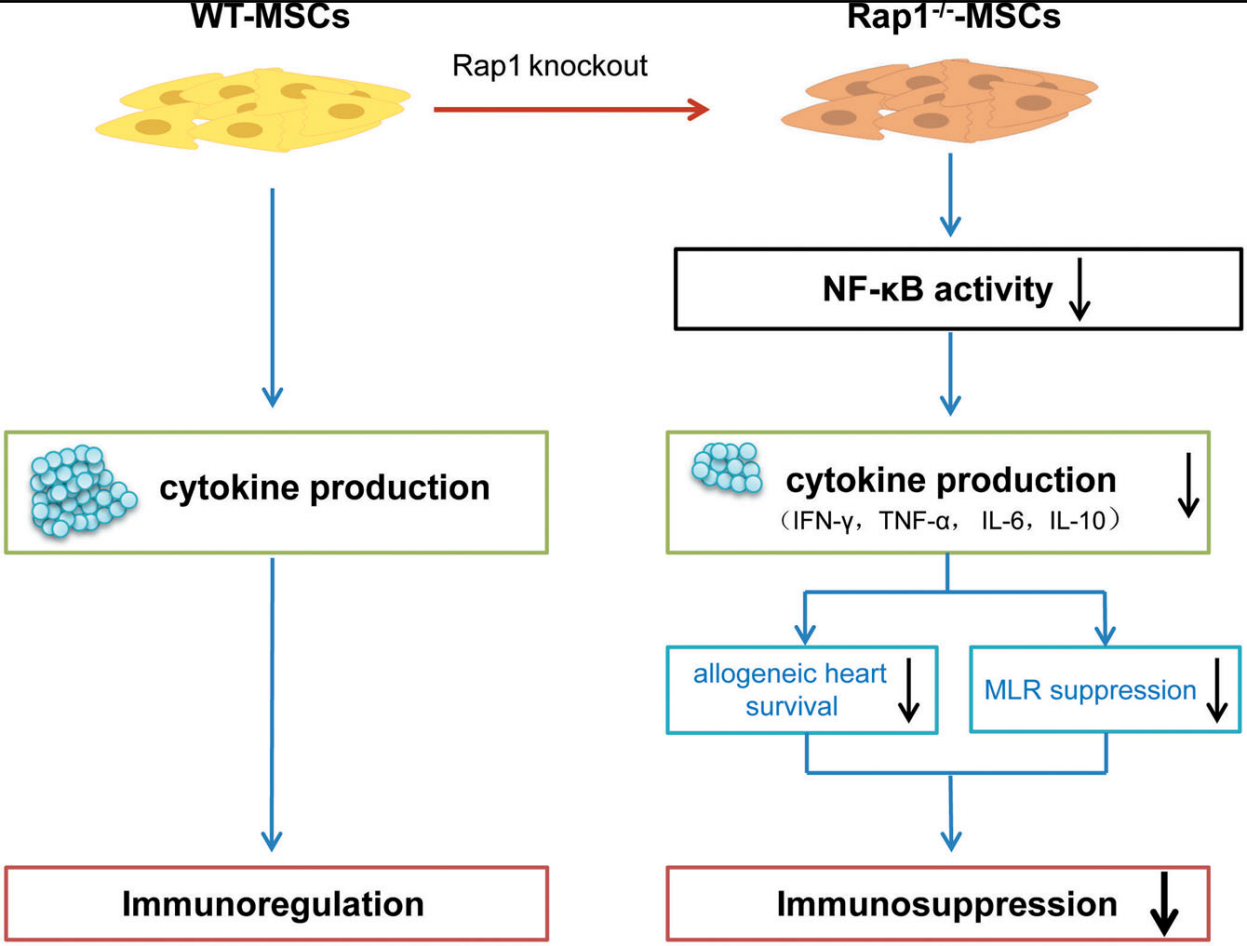
Scheme summarizing the proposed mechanism of action behind Rap1 deletion in MSC-mediated immunomodulation. The absence of Rap1 reduces the efficacy of MSCs paracrine via inhibiting the NF-κB signal pathway, which decreases the immunosuppressive potency of MSCs.

In this study, there were several major findings (Fig. 8). First, as an adjunctive therapy to RAPA, Rap1^−/−^-MSCs displayed a reduced potency to prolong the allograft survival compared with WT-MSCs, with more infiltration of inflammatory cells, less Tregs, and a higher level of proinflammatory cytokines. Second, paracrine effects exerted a leading role in MSC-mediated MLR suppression. Rap1 deficiency in MSCs resulted in a decreased capacity to inhibit MLR and reduced sensitivity to produce responsive cytokines which linked to impaired NF-κB activity. Third, by transplanting encapsulated MSCs, we demonstrated that although not as efficient as direct cell transplantation, the bioactive cytokines secreted from MSCs showed a longer allograft survival than RAPA monotherapy. The absence of Rap1 reduced the efficacy of MSC secretome in suppressing immune rejection following heart transplantation.

MSCs are highly heterogeneous and pleiotropic cells that are highly sensitive to different microenvironments, especially those containing cytokines. Although the majority of studies have demonstrated the immunosuppressive capacity of MSCs, additional investigations have shown immunostimulatory properties^7^. Given their versatility in immune modulation, MSCs have received considerable attention. A more thorough understanding of the detailed molecular mechanisms might be helpful for precise control of MSC behavior in physiological and pathological environments, and be of practical as well as fundamental value to biomedical research.

The NF-κB transcription factor family is a pleiotropic regulator involved in the control of a large number of normal cellular and organic processes, providing a mechanism for cells to respond to a wide variety of stimuli linked to inflammation^22^. Inhibition of NF-κB activation has been considered a potential therapy for inflammatory diseases. Phosphorylation of IκB proteins by the IκB kinase (IKK) complex is a crucial step in pathways and leads to NF-κB activation. It is thus logical to postulate that inhibition of IKK should have an anti-inflammatory effect by suppressing NF-κB activation. Nonetheless, in practice, IKK inhibitor treatment enhances proinflammatory IL-1β production and neutrophil accumulation that increases the susceptibility to infection^23,24^, suggesting that NF-κB also exerts an anti-inflammatory effect. Therefore, the identification of critical regulator(s) that modulate(s) immune tendentiousness in the functioning of the NF-κB signal pathway is of importance. Using a genome-wide gain-of-function screen, we identified Rap1 as a novel IKK adapter that specifically modulates NF-κB-dependent transcription in mammals by enhancing the recruitment of IKKs to the NF-κB p65 subunit^11^.

MSCs and the NF-κB pathway have countereffects with pro- and anti-inflammatory properties. This makes the results of their combination unpredictably complicated. In a mouse model of myocardial infarction (MI), we showed that the absence of Rap1 in MSCs reduced the secretion of proinflammatory cytokines and had favorable protective effects post infarction^25^. Nonetheless, in the context of allogeneic heart transplantation which arouses a violent immune response, Rap1 expression is irreplaceable for MSCs to execute immune-suppressive functions. When injected *in vivo*, Rap1^−/−^-MSCs are less efficient in downregulating proinflammatory cytokine concentration in the transplanted allograft compared with WT-MSCs, but yield comparable capacity to affect anti-inflammatory cytokines. This disrupts the fine balance between pro- and anti-inflammatory mechanisms. *In vitro*, deletion of Rap1 in MSCs leads to paracrine insufficiency: both pro- and anti-inflammatory cytokines are downregulated. Our data were partly in accordance with our previous study in which deletion of Rap1 reduced the proinflammatory factor release by MSCs^25^, but in the current study, Rap1 deficiency also reduced anti-inflammatory factors. The reduced release of anti-inflammatory cytokines might serve a predominant role rather than proinflammatory cytokines, which tilts the scale toward a proinflammatory state and leads to a decreased ability to suppress MLR in Rap1^−/−^-MSCs compared with WT-MSCs. In addition, we utilized encapsulation methods to deliver MSCs *in vivo* that allowed dynamic cytokine release without direct cell–cell contact. The effectiveness of paracrine actions in inducing allograft tolerance is affirmed, although not as evident as direct cell injection. E_Rap1^−/−^-MSCs are inferior to E_WT-MSCs in extending allograft survival, indicating that the absence of Rap1 reduces the ability of MSCs to secrete immunosuppressive factors.

The functions of MSCs are not constitutive or fixed, but rather the result of a cross talk with the microenvironment^26^. MSCs are able to sense their environment and secrete biologically active substances responsively^27^. Therefore, to harness the therapeutic potential of MSCs, signaling pathways or specific genes that hold the potential to modulate cytokine secretion should be specifically sought in different disease models. The absence of Rap1 in MSCs decreases the NF-κB sensitivity to stress-induced proinflammatory cytokine production and reduces apoptosis, and therefore benefits the therapeutic efficacy in MI^25,28^. Nonetheless, in the current study, the deficiency of Rap1 in MSCs appeared detrimental in suppressing cardiac allograft rejection. We understand the contradictory effects of Rap1 from two aspects. First, MSCs are exposed to different environments in MI and heart transplantation. The pathological characteristics of MI are multistage and intricate, involving edema, nucleomegaly, acute and chronic inflammation, granulation, and fibrotic tissue formation^29^. On the contrary, heterotopic heart transplantation mainly arouses an allograft immune response^30^. As clairvoyant as MSCs, they might act differently in accordance with the milieu to which they are exposed. Second, in the MI model, MSCs were delivered into the myocardium where dystrophy caused by ischemia seriously hampers cell survival. In the current study, we injected MSCs into the peritoneum where cell survival could be better preserved by forming aggregates in the peritoneum and producing immunoregulatory molecules^31,32^.

In summary, our study suggests that Rap1 is essential for MSCs to maintain their immunomodulatory functions. Deletion of Rap1 results in immunomodulatory impairment of MSCs. How Rap1 regulates the specificity of NF-κB signaling pathways for immunomodulatory functions of MSCs deserves further investigation.

## Materials and methods

### Animals

BALB/C and C57/B6 mice were purchased from the laboratory animal unit of the University of Hong Kong. Rap1^−/−^ and WT mice were kind gifts from the laboratory of Dr. Vinay Tergaonkar in Singapore. The handling of the animals and experimental protocols applied in this study were approved by the Committee on the Use of Live Animals in Teaching and Research (CULATR, Approval ID 2817-12) at the University of Hong Kong^33^.

### Isolation and characterization of MSCs

For MSC isolation, tibias and fibulas of Rap1^−/−^ or WT mice (6–8 weeks) were isolated and flushed using a 29-G syringe. The MSCs were cultured in complete medium that comprised DMEM/low glucose (Hyclone, SH30021.02), 15% FBS (Life Technologies, 16000), 5 ng/mL epidermal growth factor (PeProTech, AF-100-15), 5 ng/mL fibroblast growth factor (PeProTech, 100-18B), 0.1 mM 2-mercaptoethanol (Life Technologies, 21985023), Glutamax (Life Technologies, 35050061), NEAA (Life Technologies, 11140050) and penicillin–streptomycin (Life Technologies, 15140122). The isolated cells were allowed to expand until passage 4–8, and then a subset of MSCs was used for characterization as described below. The others were set aside for transplantation and *in vitro* experiments.

MSCs were characterized by surface marker profiling and their ability to differentiate into three germ layers. Cell- surface antigens for MSCs were analyzed by flow cytometry. We incubated 1.5 × 10^5^ cells with each of the following conjugated antibodies: CD105-PE (eBioscience, 12-1051-81), CD90.2-PE (eBioscience, 12-0903-82), CD73-PE (eBioscience, 12-0731-81), CD45-PE (eBioscience, 12-0451-82), CD44-APC (eBioscience, 17-0441-82), CD34-APC (eBioscience, 17-0349-42), and Sca1-APC (eBioscience, 17-5981-82). Nonspecific fluorescence was determined by incubation of similar cell aliquots with isotype-matched antibodies (eBioscience). Data were analyzed by collecting 30,000 events on a Beckman Coulter FC500 using CXP Analysis 2.0 software. For differentiation ability, adipogenesis, chondrogenesis, and osteogenesis of MSCs was determined using the relevant commercial kit according to the manufacturer’s instructions (adipogenesis: Life Technologies, A1007001; chondrogenesis: Life Technologies, A1007101; and osteogenesis: Life Technologies, A1007201). Oil red, alcian blue, and alizarin red staining for adipocytes, chondrocytes, and osteocytes, respectively, was performed using standard techniques after differentiation as previously described^34^.

### Cell proliferation assay

Cell proliferation was assayed using CCK-8 kit (CK04, Dojindo) as per the manufacturer’s protocol. Briefly, MSCs were plated into a 96-well plate and incubated with CCK-8 for 2 h at 37 °C. Absorbance was subsequently measured on a spectrophotometer at 450 nm. Three independent experiments were performed.

### Role of Rap1 in regulating NF-κB activity

Expression plasmid pAG304-Rap1 was constructed by inserting Rap1 gene containing flanked BamH1 and Xho1 restriction sites into pAG304 vector under the control of cytomegalovirus (CMV) promoter (Supplementary Fig. 2). Reporter plasmids pNF-κB-Luc and negative controls were acquired from BPS Bioscience (San Diego, CA). IκB DN was described previously^35^. Rap1^−/−^-MSCs were grown at 37 °C, and 2 × 10^5^ cells were seeded onto a 35-mm culture plate a day before transfection. Cells were transfected using Lipofectamine 2000 (Life Technologies, 11668019) with the respective constructs as described previously^36^. Cells were harvested 48 h after transfection, and the luciferase activity was determined according to the manufacturer’s instructions.

### Murine heart transplantation model

Vascularized heterotopic heart transplantation was performed from BALB/C donors to C57/B6 mice recipients by anastomosing the vessels of the neck using a non-suture cuff technique at day 0^37^. Recipient mice received rapamycin from Day 0 to Day 10 via intragastric administration unless the cardiac allografts lost contraction ability. For MSC-treated groups, mice were grouped to receive intraperitoneal injection of WT-MSCs, Rap1^−/−^-MSCs, E_WT-MSCs, or E_Rap1^−/−^-MSCs at Day 1 (1 × 10^6^/100 μL per mouse). Animals were grouped as listed in Table 1. Each group contained 16 mice, and graft condition was observed twice daily. Loss of palpable cardiac contraction was defined as complete rejection.

**Table 1:** Experimental groups

TREATMENT	GROUPS						
	Ctrl	H_RAPA	L_RAPA	L_RAPA + Rap1^−/−^-MSCs	L_RAPA + WT-MSCs	L_RAPA + E_Rap1^−/−^-MSCs	L_RAPA + E_WT-MSCs
Heart transplantation	+	+	+	+	+	+	+
RAPA (1 mg/kg/d)	−	−	+	+	+	+	+
RAPA (2 mg/kg/d)	−	+	−	−	−	−	−
Rap1^−/−^-MSCs	−	−	−	+	−	−	−
WT-MSCs	−	−	−	−	+	−	−
E_Rap1^−/−^-MSCs	−	−	−	−	−	+	−
E_WT-MSCs	−	−	−	−	−	−	+

*RAPA:* rapamycin, *Rap1*^−/−^-*MSCs:* Rap1 knockout MSCs, *WT-MSCs*: wild-type MSCs, *E_Rap1*^−/−^-*MSCs:* encapsulated Rap1^−/−^-MSCs, *E_WT-MSCs:* encapsulated WT-MSCs.

### WBC and PLT measurement

Blood was harvested from the tail vein using a capillary tube at 48 h after surgery. The number of WBC and PLT was counted using a cell counter.

### Transplanted cell engraftment in cardiac allograft

For live cell counting, the cardiac allograft was enzymatically digested at 48 h after transplantation. Dissociated cells were separated by filtering through a 30-μm filter. Most of the cardiomyocytes (>30-μm diameter) were discarded and the small cell fraction (<30 μm) was collected. The number of GFP^+^ cells was counted using FACSAria II.

### Histological evaluation of rejection

As soon as the cardiac allografts stopped beating, they were harvested and fixed with 10% PBS–formalin. Paraffin-embedded transventricular tissue sections (5 μm) were processed for hematoxylin and eosin (H&E) staining. The rejection score was determined by the extent of leucocytic infiltration and anatomical destruction of myocytes according to the criteria published by ISHLT^38^.

### Cellular phenotypic expression

Infiltrating CD45^+^ mononucleocyte population in cells isolated from transplanted hearts was examined by FACS at 48 h after surgery. For phenotypic analysis of CD4^+^Foxp3^+^ Treg cells, the cardiac allograft and spleen cells from the recipients were collected at 48 h after transplantation. CD4^+^Foxp3^+^ Treg cells were identified using an anti-mouse Foxp3-APC (eBioscience, 88-8118-40) staining set, along with a CD4-FITC (eBioscience, 11-0042-85) antibody.

### Cytokine profile *in vivo*

To distinguish the modulatory effects of Rap1^−/−^-MSCs/WT-MSCs in a cardiac transplantation model, mice serum was collected via puncture of the allograft in different groups at 48 h after surgery. The cytokines were measured using Flowcytomix (eBioscience, BMS820FFSA) as per the manufacturer’s instructions.

### *In vitro* MLR assay

To determine whether MSCs regulate the immune response, Rap1^−/−^-MSCs or WT-MSCs in gradient concentrations (2.5 × 10^4^/well; 0.25 × 10^4^/well; and 0.025 × 10^4^/well) were seeded in a 96-well round-bottom plate and cultured at 37℃ in a humidified incubator for 4 h to allow cell adherence. BALB/C donor splenocytes were prepared as stimulator cells following treatment with mitomycin C (40 μg/mL, Sigma, M0503). C57/B6 splenocytes were used as responder cells. The stimulator cells (BALB/C splenocytes) were mixed with responder cells (C57/B6 splenocytes) at a ratio of 1:10 (0.5 × 10^5^: 5 × 10^5^/well) in MLR culture medium (RPMI1640 supplemented with 10% fetal bovine serum, 1% penicillin, and streptomycin; 200 μL/well), and then added to the Rap1^−/−^-MSC/WT-MSC pre-seeded plates. Since the MSCs were seeded in gradient concentrations (2.5 × 10^4^/well; 0.25 × 10^4^/well; and 0.025 × 10^4^/well), the ratio of MSCs (5 × 10^5^/well) to responder cells was 1:20, 1:200, and 1:2000, respectively. The mixture of stimulator and responder cells was added to non-MSC plates as MLR controls. Responder cells cultured in a medium without stimulator cells served as a negative control. There were triplicate wells for each group. After 72 h of coculture, cell proliferation was quantified using a bromodeoxyuridine (BrdU) kit (Roche Applied Science, 11647229001) according to the manufacturer’s instructions.

To determine whether the paracrine effects generated by MSCs influence the MLR response, a concentrated conditioned medium collected from Rap1^−/−^-MSCs/WT-MSCs was applied to the MLR mixture. The conditioned medium (CM) was prepared as previously described.^7^ Briefly, when Rap1^−/−^-MSCs/WT-MSCs were 90% confluent on a 10-cm plate, the growth medium was aspirated, and the cells were washed three times with PBS (phosphate buffer solution), followed by the addition of 8 mL of DMEM (Gibco, 11880-036). The CM was collected after 24 h, and referred to as CM_Rap1^−/−^-MSCs or CM_WT-MSCs (Fig. 5b-i). Since MSC secretome may differ under inflammatory conditions compared with the normal physiological environment, the supernatant of activated lymphocytes, referred to as CM_MLR, was prepared by coculturing stimulator cells (BALB/C splenocytes) and responder cells (C57/B6 splenocytes) at a ratio of 1:10 for 96 h. Then, CM_MLR was added to Rap1^−/−^-MSCs/WT-MSCs as an inducer and cultured for 24 h. CM_MLR was aspirated, and the cells were washed three times with PBS prior to the addition of 8 mL of DMEM (Gibco, 11880-036). The supernatant was collected after a further 24 h to allow efficient cytokine release, and referred to as iCM_Rap1^−/−^-MSCs or iCM_WT-MSCs. The experimental schematic diagram is depicted in Fig. 5b-ii. After collection, the CM was cleared by centrifugation, filtered through a 0.22-μm Millipore filter, and then placed into an Amicon Ultra-4 Centrifugal Filter Unit (Millipore, UFC801024) for purification and concentration according to the manual with a concentration factor of 10 before application in the MLR assay. After 72 h of coculture, cell proliferation was quantified by BrdU assay.

### Cytokine profiling *in vitro*

CM_WT-MSCs, CM_Rap1^−/−^-MSCs, iCM_WT-MSCs, and iCM_Rap1^−/−^-MSCs were collected, concentrated as described above, and quantified by Bradford assay (BIO-RAD, 5000202). The secreted level of IFN-γ, TNF-α, IL-10, IL-6, and TGF-β was measured using a bead-based analyte detection System-Flowcytomix (eBioscience, BMS820FFSA) according to the manufacturer’s instructions.

### Restoring Rap1 expression in Rap1^−/−^-MSCs

Rap1 overexpressing plasmid was purchased from TAKARA. Transient transfection was performed using Lipofectamin 2000 (Thermo Scientific, 11668019) according to the manufacturer's instructions. At 48 h after transfection, Rap1 expression was measured via western blotting analysis.

### QNZ treatment

QNZ was purchased from Sellectchem (S4902). Rap1^−/−^-MSCs, Rap1^+/+^-MSCs, and WT-MSCs were cultured in T25 until they reached 85–90% confluency, and then CM_MLR was added as an inducer and cultured for 24 h. CM_MLR was aspirated, and the cells were washed three times with PBS prior to the addition of 5 mL of DMEM (Gibco, 11880-036) and QNZ (11 nM). After 24 h of incubation, the supernatant was collected and concentrated. The secreted level of IFN-γ, TNF-α, IL-6, and IL-10 was measured using a bead-based analyte detection System-Flowcytomix (eBioscience, BMS820FFSA), according to the manufacturer’s instructions. Cells were harvested and used for western blotting analysis.

### Western blotting

Cells were harvested and lysed in RIPA buffer (Sigma, R0278) supplemented with complete ULTRA tablets (ROCHE, 5892970001). The protein concentrations were measured by BCA protein assay kit (Thermo Scientific Pierce, 23235). A total amount of 30 μg of protein from each sample was loaded. Samples were separated by SDS/PAGE and then transferred to a PVDF membrane. Membranes were blocked in 5% wt/vol fat-free milk (BIO-RAD, 170-6404) and then incubated with the following primary antibodies overnight at 4 °C: Rap1 (Thermo Scientific, PA5-27588), GAPDH (eBioscience, 14-9523-80), NF-κB p65 (Abcam, ab32536), and pNF-κB p65 (Abcam, ab194921). After washing, membranes were incubated with appropriate HRP-conjugated secondary antibodies at a 1:1000 dilution. Bands were detected using enhanced chemiluminescence (GE Healthcare, RPN2232).

### Preparation of encapsulated MSCs

For the cell encapsulation experiment, 2.0% (w/w) sodium alginate (Aladdin Chemistry Co., Ltd., China) dissolved in distillation–distillation water was prepared as the precursor solution by magnetic stirring for 1 h. A volume of 3.0 wt.% calcium chloride (Wing Hing Chemical Co., Ltd., Hong Kong) solution was prepared and added to a collection bath. Afterward, MSCs were mixed with the precursor solution to form a cell suspension with a density of 1 × 10^9^ cells/mL.

To achieve cell-encapsulated hydrogel particles with controlled sizes, we electrosprayed the liquid jet under a controlled electric field. When a high voltage was applied, the liquid jet was stretched by the electric stress and assumed a tapered shape. Next, the tapered jet broke up into uniform alginate droplets that were collected in a bath of calcium chloride solution. The alginate droplets were subsequently cross-linked by calcium ions and formed calcium alginate hydrogel particles.

The number of cells per particle could be manipulated by varying the density of the cells in the precursor suspension; the size of the particles was in turn controlled by the magnitude of the electric field. In this study, with an applied electric field of 3.2 × 10^5^ V/m, the average diameter of the particles was 206 μm; each particle contained an average of 4580 cells.

### Statistical analysis

All data are presented as the mean ± standard error of mean (SEM). Allograft survival curves were plotted using the Kaplan–Meier method, and the difference in allograft survival among groups was compared using the log-rank test. A comparison of parameters between two groups was performed by unpaired Student’s *t*-test. Comparison of variables between multiple groups was performed using one-way ANOVA with the Bonferroni post hoc test. Calculations were performed with SPSS 16.0 and *p* < 0.05 was considered statistically significant.

## Electronic supplementary material

supplementary information

supplementary Figure 1

supplementary Figure 2

## References

[1] Reinders MEJ (2013). Autologous bone marrow-derived mesenchymal stromal cells for the treatment of allograft rejection after renal transplantation: results of a phase I study. Stem Cell Transl. Med..

[2] Muroi K (2013). Unrelated allogeneic bone marrow-derived mesenchymal stem cells for steroid-refractory acute graft-versus-host disease: a phase I/II study. Int. J. Hematol..

[3] Eggenhofer E (2011). Features of synergism between mesenchymal stem cells and immunosuppressive drugs in a murine heart transplantation model. Transpl. Immunol..

[4] Cheng PP (2015). Mesenchymal stem cells derived from induced pluripotent stem cells combined with low-dose rapamycin induced islet allograft tolerance via suppressing Th1 and enhancing regulatory T cell differentiation. Stem Cells Dev..

[5] Sudres M (2006). Bone marrow mesenchymal stem cells suppress lymphocyte proliferation in vitro but fail to prevent graft-versus-host disease in mice. J. Immunol..

[6] Ringden O (2006). Mesenchymal stem cells for treatment of therapy-resistant graft-versus-host disease. Transplantation.

[7] Li W (2012). Mesenchymal stem cells: a double-edged sword in regulating immune responses. Cell Death Differ..

[8] Di Nicola M (2002). Human bone marrow stromal cells suppress T-lymphocyte proliferation induced by cellular or nonspecific mitogenic stimuli. Blood.

[9] Lawrence T (2009). The nuclear factor NF-kappaB pathway in inflammation. Cold Spring Harb. Perspect. Biol..

[10] Mutt SJ (2012). Inhibition of cytokine secretion from adipocytes by 1,25-dihydroxyvitamin D(3) via the NF-kappaB pathway. FASEB J..

[11] Teo H (2010). Telomere-independent Rap1 is an IKK adaptor and regulates NF-kappaB-dependent gene expression. Nat. Cell Biol..

[12] Poon MW (2015). Inhibition of RAP1 enhances corneal recovery following alkali injury. Invest. Ophthalmol. Vis. Sci..

[13] Ge W (2009). Infusion of mesenchymal stem cells and rapamycin synergize to attenuate alloimmune responses and promote cardiac allograft tolerance. Am. J. Transplant..

[14] Murgia MG, Jordan S, Kahan BD (1996). The side effect profile of sirolimus: a phase I study in quiescent cyclosporine-prednisone-treated renal transplant patients. Kidney Int..

[15] Lin Y (2011). Arsenic trioxide is a novel agent for combination therapy to prolong heart allograft survival in allo-primed T cells transferred mice. Transpl. Immunol..

[16] Liang X, Ding Y, Zhang Y, Tse HF, Lian Q (2014). Paracrine mechanisms of mesenchymal stem cell-based therapy: current status and perspectives. Cell Transplant..

[17] Sica A (1997). Interaction of NF-kappaB and NFAT with the interferon-gamma promoter. J. Biol. Chem..

[18] Collart MA, Baeuerle P, Vassalli P (1990). Regulation of tumor necrosis factor alpha transcription in macrophages: involvement of four kappa B-like motifs and of constitutive and inducible forms of NF-kappa B. Mol. Cell. Biol..

[19] Son YH (2008). Roles of MAPK and NF-kappaB in interleukin-6 induction by lipopolysaccharide in vascular smooth muscle cells. J. Cardiovasc. Pharmacol..

[20] Cao S, Zhang X, Edwards JP, Mosser DM (2006). NF-kappaB1 (p50) homodimers differentially regulate pro- and anti-inflammatory cytokines in macrophages. J. Biol. Chem..

[21] Goren A, Dahan N, Goren E, Baruch L, Machluf M (2010). Encapsulated human mesenchymal stem cells: a unique hypoimmunogenic platform for long-term cellular therapy. FASEB J..

[22] Pires, B. R. B., Silva, R., Ferreira, G. M. & Abdelhay, E. NF-kappaB: two sides of the same coin. *Genes* **9** (2018). 10.3390/genes9010024 PMC5793177 29315242

[23] Greten FR (2007). NF-kappaB is a negative regulator of IL-1beta secretion as revealed by genetic and pharmacological inhibition of IKKbeta. Cell.

[24] Hsu LC (2011). IL-1beta-driven neutrophilia preserves antibacterial defense in the absence of the kinase IKKbeta. Nat. Immunol..

[25] Zhang Y (2015). Rap1-mediated nuclear factor-kappaB (NF-κB) activity regulates the paracrine capacity of mesenchymal stem cells in heart repair following infarction. Cell Death Discov..

[26] Gao, F. et al. Mesenchymal stem cells and immunomodulation: current status and future prospects. *Cell Death Dis.* **7** (2016). 10.1038/cddis.2015.327 PMC4816164 26794657

[27] Le Blanc K, Mougiakakos D (2012). Multipotent mesenchymal stromal cells and the innate immune system. Nat. Rev. Immunol..

[28] Poon MW (2015). Inhibition of RAP1 enhances corneal recovery following alkali injury. Invest. Ophth. Vis. Sci..

[29] Salto-Tellez M (2004). Myocardial infarction in the C57BL/6J mouse: a quantifiable and highly reproducible experimental model. Cardiovasc. Pathol..

[30] Liu, F. & Kang, S. M. Heterotopic heart transplantation in mice. *J. Vis. Exp.* **238** (2007). 10.3791/238 PMC2557111 18997886

[31] Sala E (2015). Mesenchymal stem cells reduce colitis in mice via release of TSG6, independently of their localization to the intestine. Gastroenterology.

[32] Liu L (2014). Intranasal versus intraperitoneal delivery of human umbilical cord tissue-derived cultured mesenchymal stromal cells in a murine model of neonatal lung injury. Am. J. Pathol..

[33] (CULATR) C.f.t.U.o.L.A.i.T.a.R. http://www.med.hku.hk/v1/research/research-ethics/animal-ethics-culatr (2014).

[34] Meirelles Lda S, Nardi NB (2003). Murine marrow-derived mesenchymal stem cell: isolation, in vitro expansion, and characterization. Br. J. Haematol..

[35] Nakano H (1998). Differential regulation of IkappaB kinase alpha and beta by two upstream kinases, NF-kappaB-inducing kinase and mitogen-activated protein kinase/ERK kinase kinase-1. Proc. Natl Acad. Sci. USA.

[36] Poon MW (2017). Inhibition of NUCKS facilitates corneal recovery following alkali burn. Sci. Rep..

[37] Matsuura A, Abe T, Yasuura K (1991). Simplified mouse cervical heart transplantation using a cuff technique. Transplantation.

[38] Billingham ME (1990). A working formulation for the standardization of nomenclature in the diagnosis of heart and lung rejection: Heart Rejection Study Group. The International Society for Heart Transplantation. J. Heart Transplant..

