# Supplementary material for: Rap1 deficiency-provoked paracrine dysfunction impairs immunosuppressive potency of mesenchymal stem cells in allograft rejection of heart transplantation
Source: Cell Death Dis. 2018 Mar 7;9(3):386. doi: 10.1038/s41419-018-0414-3 (PMC5842217; doi:10.1038/s41419-018-0414-3)
Supplement: Supplementary file 1 — supplementary information [file 41419_2018_414_MOESM1_ESM.docx]

**Rap1 deficiency-provoked paracrine dysfunction impairs immunosuppressive potency of mesenchymal stem cells in allograft rejection of heart transplantation**

Yue Ding, MD^1,5,8,#^; Xiaoting Liang, MD, PhD^2,3,5,#^; Yuelin Zhang, MD, PhD^4,5^; Li Yi, MD^6^; Ho Cheung Shum, PhD^9^; Qiulan Chen, PhD^9^; Barbara P.Chan, PhD^9^; Huimin Fan, MD, PhD^11^; Zhongmin Liu, MD, PhD^2,11^; Vinay Tergaonkar, PhD^10^; Zhongquan Qi , MD, PhD^8*^; Hung-fat Tse, MD, PhD^5*^; Qizhou Lian, MD, PhD^5,6,7*^

^1^Department of Organ Transplantation, Changzheng Hospital, Second Military Medical University, Shanghai, P.R. China;

^2^Translational Medical Center for Stem Cell Therapy, Shanghai East Hospital, Tongji University School of Medicine, Shanghai, P.R. China;

^3^Clinical Translational Medical Research Center, Shanghai East Hospital, Tongji University School of Medicine, Shanghai, P.R. China;

^4^Department of Emergency, Guangdong General Hospital, Guangdong Academy of Medical Sciences, Guangzhou, P.R. China;

^5^Department of Medicine, the University of Hong Kong, Hong Kong SAR, P.R. China;

^6^Peking University Shenzhen Hospital, Shenzhen P.R. China;

^7^School of Biomedical Sciences, the University of Hong Kong, Hong Kong SAR, P.R. China;

^8^Organ Transplantation Institute of Xiamen University, Xiamen, Fujian Province, P.R. China;

^9^Department of Mechanical Engineering, the University of Hong Kong, Hong Kong SAR, P.R. China;

^10^Institute of Molecular and Cellular Biology, Biopolis, Singapore;

^11^Department of Cardiovascular and Thoracic Surgery, Shanghai East Hospital, Tongji University School of Medicine, Shanghai, P.R. China;

^#^These authors contribute equally to this work.

*Correspondence:

Email: [qzlian@hku.hk](mailto:qzlian@hku.hk) (Qizhou Lian); [hftse@hku.hk](mailto:hftse@hku.hk) (Hung-fat Tse); [oti@xmu.edu.cn](mailto:oti@xmu.edu.cn) (Zhongquan Qi)

**Running Title:** Rap1-NFκB pathway regulates MSC-mediated immunomodulation

**Figure legend**

**Supplementary Figure 1.** Characterization of MSCs. A) Surface marker profiling of Rap1^-/-^-MSCs and WT-MSCs. Both Rap1^-/-^-MSCs and WT-MSCs were positive for MSC marker CD105, CD90.2, CD73, CD44 and Sca1. B) Both Rap1^-/-^-MSCs and WT-MSCs retained typical spindle-like morphology *in vitro*, and possessed multiple differentiation ability for adipocyte, chondrocyte and osteocyte. Scale bar=100μM. C) Cell proliferation determined by CCK8 assay.

**Supplementary Figure 2.** Plasmid used for Rap1 overexpression in Rap1^-/-^-MSCs.
