# Supplementary figures and images for: Rap1 deficiency-provoked paracrine dysfunction impairs immunosuppressive potency of mesenchymal stem cells in allograft rejection of heart transplantation
Source: Cell Death Dis. 2018 Mar 7;9(3):386. doi: 10.1038/s41419-018-0414-3 (PMC5842217; doi:10.1038/s41419-018-0414-3)

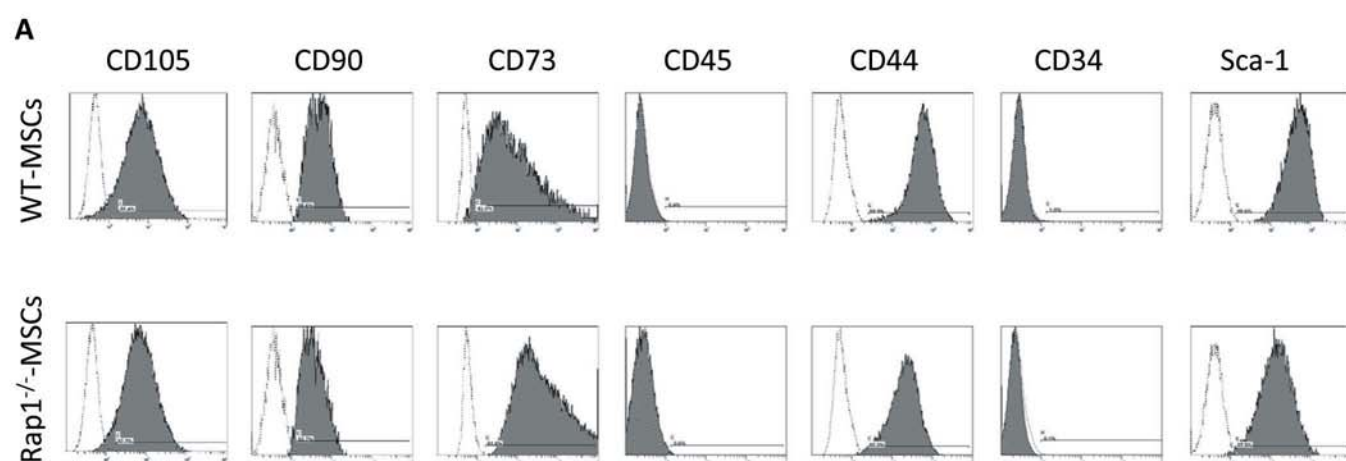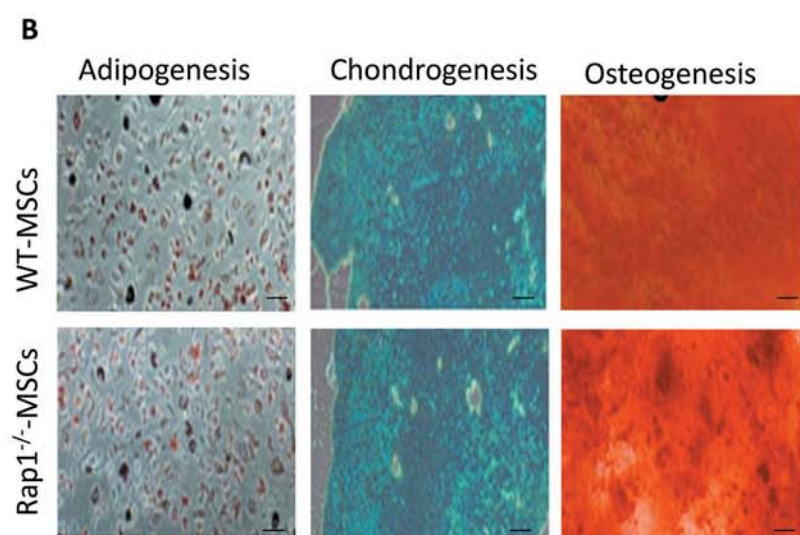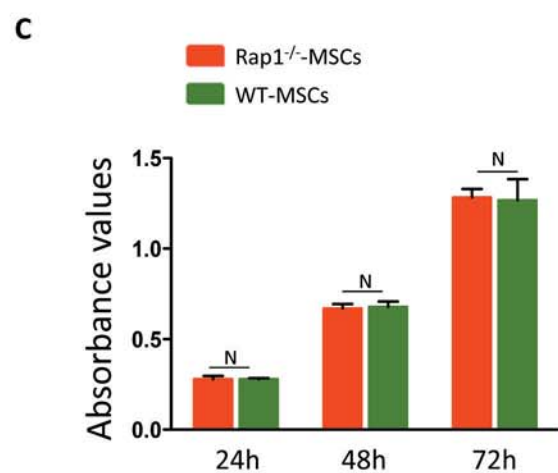

**Supplementary Figure 1**

Supplement: Supplementary file 2 — supplementary Figure 1 [file 41419_2018_414_MOESM2_ESM.pdf]

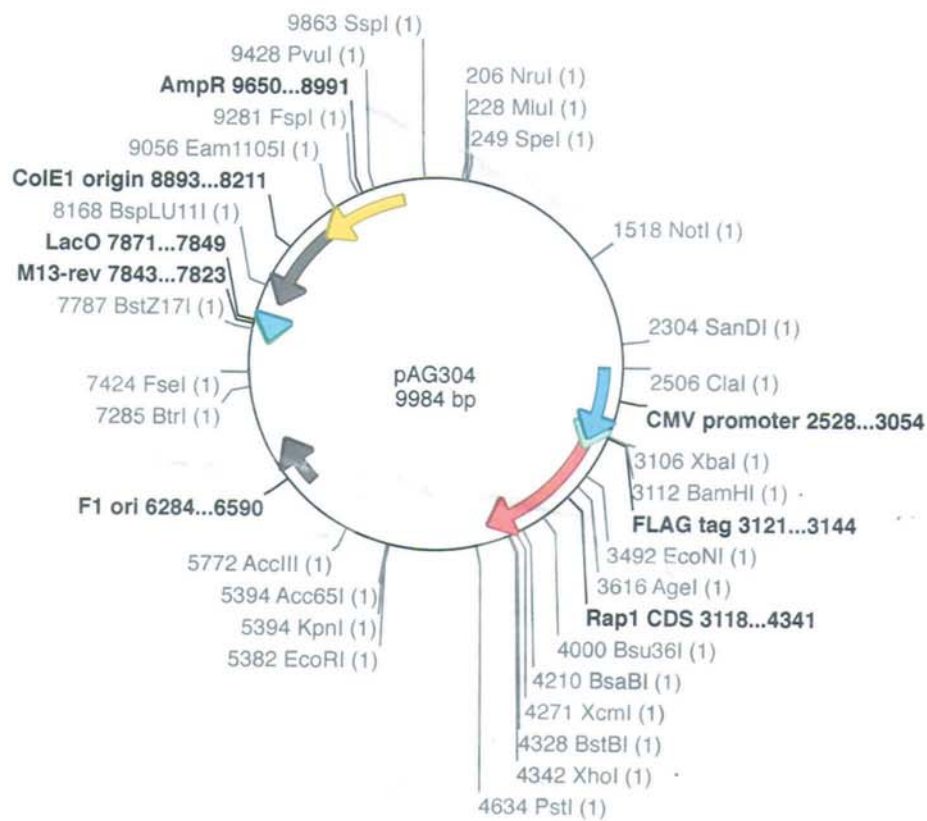

**Supplementary Figure 2**

Supplement: Supplementary file 3 — supplementary Figure 2 [file 41419_2018_414_MOESM3_ESM.pdf]
